# When a notification at the right time is not enough: the reminding process for socially assistive robots in institutional care

**DOI:** 10.3389/frobt.2024.1369438

**Published:** 2024-05-01

**Authors:** Matthias Rehm, Antonia L. Krummheuer

**Affiliations:** ^1^ Technical Faculty of IT and Design, Aalborg University, Aalborg, Denmark; ^2^ Faculty of Social Sciences and Humanities, Aalborg University, Aalborg, Denmark

**Keywords:** social robots, reminder, cognitive impairment, human robot interaction, practice

## Abstract

Reminding is often identified as a central function of socially assistive robots in the healthcare sector. The robotic reminders are supposed to help people with memory impairments to remember to take their medicine, to drink and eat, or to attend appointments. Such standalone reminding technologies can, however, be too demanding for people with memory injuries. In a co-creation process, we developed an individual reminder robot together with a person with traumatic brain injury and her care personnel. During this process, we learned that while current research describe reminding as a prototypical task for socially assistive robots, there is no clear definition of what constitutes a reminder nor that it is based on complex sequences of interactions that evolve over time and space, across different actions, actors and technologies. Based on our data from the co-creation process and the first deployment, we argue for a shift towards a sequential and socially distributed character of reminding. Understanding socially assistive robots as rehabilitative tools for people with memory impairment, they need to be reconsidered as interconnected elements in institutional care practices instead of isolated events for the remindee.

## 1 Introduction

Healthcare robots are seen as promising solutions to central challenges of our healthcare systems. The demographic challenge of a fast-growing ageing population puts a severe strain on both the human and the financial resources required to maintain a high quality of health and care. A central challenge is the increasing number of people with cognitive challenges that can be caused by, e.g., acquired brain injury (ABI) (2.5 million people suffer each year in Europe, from which 1.5 million have to be hospitalized; 120.000 people in Denmark are living with ABI)^1^, Alzheimer or dementia (85.000–90.000 people in Denmark are estimated to have dementia, 7.85 million in Europe)^2^, or other age-associated memory impairments. These cognitive impairments often impact the person’s participation in and organisation of activities of daily living (ADL) (e.g., maintaining house hold chores and managing appointments) with psychological and social consequences, “since forming and maintaining relationships depends partially on the ability to make and keep dates for social activities, recall information about others’ likes and dislikes, and discuss previous shared interactions” (Sander and van Veldhoven, 2014, p. 174). People with cognitive impairment are therefore dependent on an assistive network supporting them in their everyday lives. Technological innovations such as socially assistive robots (SARs) are seen as promising solutions with its ambition for developing care to empower (elderly) citizens, increase their self-sufficiency, and improve their quality of life while at the same time using human and financial resources most efficiently (Matarić et al., 2009; Matarić and Scassellati, 2016; Uddannelses-og Forskningsministeriet, 2020). In surveys on the use of SARs in healthcare contexts, reminding, and especially reminding in relation to medication and upcoming ADL, is described as a prototypical example where such robots could be employed (e.g., Petrie and Darzentas, 2017; Martinez-Martin and del Pobil, 2018; Shishehgar et al., 2018; Juel et al., 2020; Khan and Anwar, 2020; Lee and Naguib, 2020; Mois and Beer, 2020).

In line with this argument, we engaged in a co-creation process in which we developed and implemented a robotic reminding system for and together with people with acquired brain injury and their care personnel (Rehm et al., 2018; Rodil et al., 2018). However, in one of our cases despite several iteration in the design process, the reminder function of the robot was not accepted when tested in practice. This failure was the consequence of a hidden assumption during the design process that resulted in the neglect of a) the situated, interactive construction of reminders in supportive healthcare settings, b) the organisational shaping of reminders over time and their individual adjustments, b) misunderstandings of the concept of autonomy in context of severe memory problems and d) a wrongly assumed dyadic understanding of human-robot interaction. This paper reports our change of perspective and summarizes central insights that we hope will prevent future projects to make the same mistakes. The argument is based on a systematic exploration and reflection of our video data that we collected for documentation of our co-creation process but not for a systematic analysis of a reminding practices. Therefore it follows its own structure. We will start with a brief description of the robot system that failed and the co-creation process in which it was developed. Then we will describe the practices of reminding based on the video data from the workshops and review recent studies on the development of reminder robots which shows that most of them have the same blind spots as we did. The article presents a new model for understanding and implementing reminder processes in SARs and ends by formulating five requirements for the future design of reminder robots.

## 2 Co-creation of a reminder robot

The insights of this paper derive from an interdisciplinary project, bringing together researchers from sociology, design, and engineering with the goal of co-creating individual guiding and reminding robots for and together with seven people living with ABI. The project was situated at a Danish residential home for people living with ABI, that also provided staff to support the process and ensure integration of the robots into the institutional routines.

In our first meetings with the residential home, we saw a collection of unused technologies that were introduced to the residence by external actors, but ended in storage rooms as they did not meet the everyday needs of residents and staff. Therefore we turned to participatory design engaging in a bottom-up process with no prior system in mind. The broad field of participatory design (PD) is committed to “ensuring that those who will use information technologies play a critical role in their design” (Robertson and Simonsen, 2012, 2). As such PD is directed to shaping future situations in a mutual learning process. The principal roles of PD are users and designers “where the designers strive to learn the realities of the user’s situation while the users strive to articulate their desired aims and learn appropriate technological means to obtain them” (Robertson and Simonsen, 2012, 2). Sanders and Stappers, 2008, differentiate between co-creation and co-design. While co-creation refers to “any act of collective creativity”, co-design is defined as “the collective creativity as it is applied across the whole span of a design process”, including not only the creativity of the designer, but also people not trained in design working together in the design development process. Similar to co-design we aimed to include all participants, that is researchers, people with ABI, and their care personnel across the span of the whole design process not only learning about participants realities but also cooperating in the material creation of a concrete robot system. However, we prefer the term of co-creation as it highlights the material co-construction of a robot during the process.

The project idea was framed by two weeks of ethnographic observation (Blomberg and Karasti, 2012; Randall and Rouncefield, 2018), in which all researchers followed different residents in their everyday activities such as grocery shopping (Krummheuer 2020, 2021) and morning routines. We also took part in joint activities such as bowling and gaming, presented robots from the university to the residents, and we held a workshop on programming to see if there is any interest in these technologies. These activities ensured us a better understanding of the residents’ everyday practices and demands, and allowed the residents and care personnel to get to know (and trust) us, and learn about our research and interest. The process resulted among others in the joint formulation of the co-creation project presented in this article.

**FIGURE 1:**
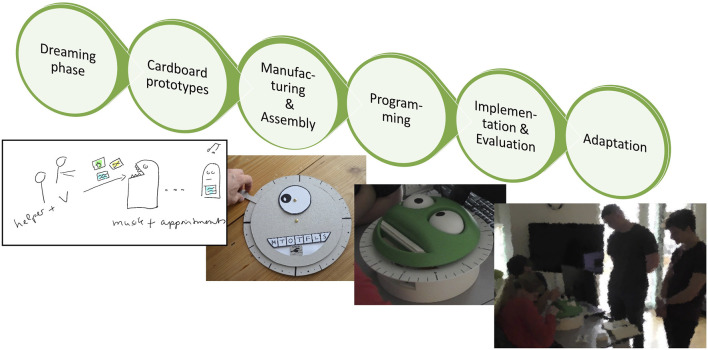
The iterative phases of the co-creation process.

The co-creation process was organised as bottom-up process in six iterative phases with no prior system in mind (see Figure 1). We started by co-exploring the residents’ everyday practices and needs and engaged in a ‘dreaming phase’ to develop first ideas of the future robot’s function and looks that were documented by sketches. In phase two, we co-created cardboard prototypes to test basic functions and looks of the robots. In phase three, we manufactured the robot’s parts, invited the residents to Aalborg University to witness the production of robot parts (e.g., 3D printing) and assembled the parts later with the residents in their apartments. This was followed and partly overlapped by the programming of the robot in phase four. Finally, we delivered the robots to the residents for daily use. All phases included several iterations of testing and evaluation in order to adapt the robot to the needs and wishes of the users (residents and sometimes staff) as well as adapting these wishes to the technical constraints imposed by the hard- and software.

The whole process took place over 18 months with monthly workshops with residents and care personnel. We approached the people with ABI as co-designers and equal partners with individual communicative and cognitive resources. To meet the needs of the participants with ABI, workshops were held only with one resident at a time, and did not proceed longer than 45 min. All residents were accompanied by care personnel who assisted them or us in understanding each other and provided relevant knowledge of organisational or individual routines. We created a folder for each resident documenting the process with pictures and descriptions of each workshop. The folder was updated after each workshop and was meant as memory help for them as well as support to talk about their experiences with family and friends. We approached the staff as co-designers, informants and potential future co-users of the robot, as we understood them as future facilitators, moderators or collaborators in the resident-robot interaction. Similar to the tell-make-enact diagram from Brandt et al. (2012), we aimed to engage participants in various ways in the co-creation process. Beside verbal descriptions and reflections, we engaged residents and staff in the making and enactment of the different prototypes.

In the remainder of this article, we report on one of the cases, in which we experienced the co-creation robot system to fail. Ida’s^3^ short-term memory is impaired due to her brain injury. This means that she is not able to remember the events of a day or week. She is also not able to understand calendar entries, as this would require to know what time and day it is, and what to do to participate in an event shown in a calendar. Therefore, she and the staff asked for a reminder robot that should not remind her of an appointment, but provide Ida with contextual clues, to prepare her for an upcoming interaction. One of her carers formulated that this ‘pre-reminder’ should provide her with “a sense of what will happen, when I come in, because Ida has forgotten what we talked about.”

**FIGURE 2:**
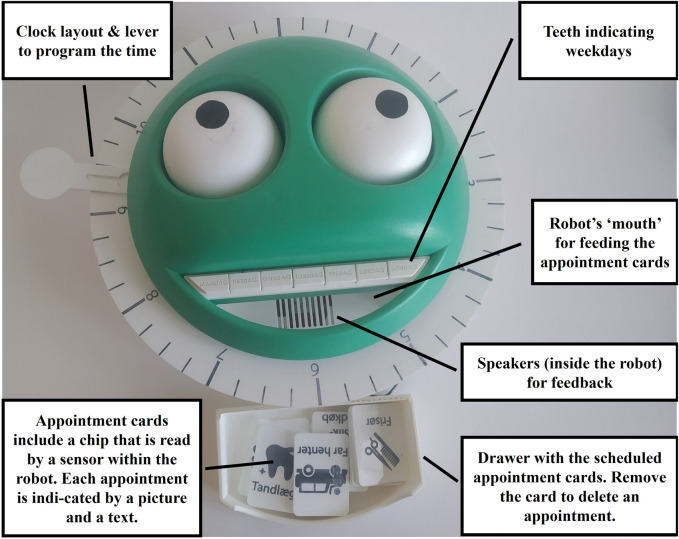
Ida’s reminding robot.

Over the course of 17 workshops a green-faced reminder robot was developed (Figure 2). The reminder was envisioned as coming in the form of music. In addition, the robot allowed Ida to program the reminder of the robot together with the staff. The resulting robot is employing a range of different interactions techniques, combining near field communication with buttons and levers for the reminder programming.^4^ Appointments are programmed by feeding the robot appointment cards (Figure 2). Each appointment card represents one regular appointment that is visualised with pictures and words, as, e.g., physiotherapy, sweet purchase and swimming. Each card includes a chip that can be read by the robot. The day of the appointment is programmed by pressing one of the robots teeth. The teeth indicate the weekdays from Monday to Sunday. The time of the appointment is programmed by placing a lever on the clock-like design around the robot’s head.

For testing the reminder robot, we asked Ida and a close carer to program the appointments of the upcoming week during a workshop. We then installed the robot in Ida’s apartment and agreed that we would come back in two weeks time to see how the robot is working, and what could be improved. Already the next day we were contacted by the staff who told us that they unplugged the robot. The robot was working well, it started to play the music to remind Ida that she would be going to physiotherapy soon. Ida was, however, confused about the music and could seemingly not remember why this music was playing. She got so agitated that she was not able to go to physiotherapy.^5^

This failure made us review the videos from all workshops with Ida. We found that the decision to base the reminder on music was a valid result of the co-creation processes. The wish to use music was formulated by Ida and the carer already early in the process and we engaged in several activities with both Ida and care personnel that confirmed this choice. The choice was based on the staff’s observation that Ida loves music, which was expressed in several workshops from different personnel. It was confirmed by Ida expressing her wish and fondness for music during the workshops, for example, often she started to smile and sing when music was played and to make sure to find the right music, she engaged in a longer selection process of finding the right song supported by her care personnel outside time frames of the workshops. Furthermore, the test of the reminder function during the workshop activities resulted in positive and confirming reactions by all participants, singing, nodding, smiling and positive comments.

Therefore, we must have overseen a taken-for-granted assumption that does not meet the practical requirements. This made us shift our theoretical lens turning to ethnomethodology, which enabled us to analyze the videos of the co-creation process with a perspective on reminding as a social practice (Section 3). Additionally, we were interested in understanding in more depth how newer studies on reminding robots define and realize reminders (Section 4).

## 3 Shifting the perspective—the dynamic and interactive nature of reminders

### 3.1 Interactive perspectives on remembering and reminding

Classical cognitive and psychological approaches understand memory as an “archive” i.e. as an individual’s capacity to store memories (Brockmeier, 2017). Within memory rehabilitation, Wilson (2009) classifies memory in relation to time as “short-term” and “long-term” memory, depending on how long an information can be stored; or “retrospective” and “prospective memory”, which affects the individual’s ability to remember the past or to remember future appointments. Within this framework a reminder is a signal that supports our capacity to retrieve a memory. In contrast, interactional or practice-oriented approaches as, for example, ethnomethodology, conversation analysis or discourse psychology conceptualise memory and cognition as situated and interactional constructions within social practices (Reckwitz, 2002; Molder and Potter, 2005; Lynch, 2006; van Dijk, 2006; Brockmeier, 2017). In this view, “mental patterns are not the ‘possession’ of an individual ‘deep inside’, but part of the social practice” (Reckwitz, 2002, p. 252). Thus, not the individual’s capacity to remember is of interest, but the interest is directed to a) the mental and social activities that constitute a practice (e.g., reminding), and b) communicative constructions of memory or cognitive activities by the participants in interaction or as activities of co-remembering.

These interactional studies describe the sequential and multimodal construction of memory and remembering in interaction. They analyse how speakers reference events and memories they presume to be shared by their co-participants, for example, in form of recognition checks such as “(do you) remember X” (You, 2015; Bolden and Mandelbaum, 2017). These recognition checks are used to establish alignment and a common ground of knowledge in interaction to support the speaker’s course of action. Equally important are the recipient’s reactions to these formulations (You, 2015). The recipient’s display or lack of assumed knowledge is made available in interaction by utterances such as “I do remember/do not remember”. The production and recognition of the reminder are thus sequentially linked and become a publicly visible display that is used by the participants to coordinate their activities. In contrast to these studies, the care personnel in our context cannot assume a shared memory due to Ida’s injury. The question is therefore how reminding is organised in interactions with Ida.

We turned to ethnomethodology to describe the sequential and multimodal construction of reminding in the workshop interaction. Ethnomethodology assumes that the taken-for-granted social order of everyday or organisational life is an situated and interactive achievement by the members of the society or setting. Meaning and social order do not just happen but are situated and public co-constructions. Ethnomethodology aims to describe the routinized, “seen but unnoticed” (Garfinkel, 1964, 226), everyday methods and expectations by which participants are able to observe, produce and recognise “what is happening around them, and thereby know what they should do to fit their action together with the actions of others” (Francis and Hester, 2004). This is done by detailed observation often using video recording to describe the sequential and multimodal unfolding of activities and the interactional work done to achieve them. This can be done, e.g., by ethnographic description mapping the sequential order of an activity. Through the use of fieldnotes and other resources we describe “the sequential order of work and the sequential accomplishment of the particular activities in and through which that sequential order is animated and produced” (Crabtree et al., 2012, 105). In the following, we use ethnographic description derived from the video data of the workshops for mapping how the robot’s reminder was embedded and constructed in the interactional organisation of the workshop when programming and testing the robot.

### 3.2 Doing reminding during workshop trials

In difference to the isolated situation where Ida received the reminder that lead to the failure of it, the tests of the robot’s reminder function during the workshops did not disrupt Ida, but it was interactively constructed as “an expected event”. During the workshops, the participants (researchers and staff) engaged in interactional work that built the context to understand the music as a reminder. This was done by introducing the structure of the workshop, explaining the robot’s function and looks, as well as describing upcoming activities, e.g., with sentences like “today you will program the robot and test if the reminder is working”, at the beginning of the workshop, and sentences like “are you ready to program the robot”, when the robot was set up. The participants thus engaged in the cooperative construction of meaning demonstrating verbally and visually to each other what they were doing and creating expectations of what will happen next. After the robot was programmed, the participants presented themselves as “waiting-for-the-reminder”, e.g., by engaging in small-talk or joking, meanwhile their bodies were not engaged in other activities (e.g., programming the robot) but directed to the robot indicating the expectation of an upcoming event and marking the robot a central piece of this expectation. When the reminder was given, it was welcomed as an awaited and positive event, e.g., with laughter, positive comments and thumb-up gestures. During these interactions, Ida aligned her actions, e.g., by waiting and laughing together with the others and starting to sing and smile when the reminder was given and thus joining the collaborative assessment of the robot. Of course, we cannot determine, whether Ida actually understood the whole context (but this can neither be done for the other participants). But we can see, that Ida could exploit the interactive framework given by the others that enabled her to take part in the ongoing interaction, in which the music was treated as an expected and meaningful event.

Elsewhere, we show that the remembering was equally co-organised in the interaction between staff and Ida when programming the robot (Krummheuer et al., 2020; Hornecker et al., 2022). In a ethnomethodological conversation analysis of video data from the programming of the robot during the workshop, we demonstrate how the carer provides an interactional framework that is exploited by Ida (see also Goodwin, 1995; 2003; Barnes, 2012; Krummheuer, 2015). For example, the carer announces the appointment in the beginning of the programming, providing the necessary information for programming the robot (appointment, day and time) and inviting Ida to program the robot. In addition, the caregiver breaks the task of programming the robot down into smaller units directing Ida’s attention first to the programming appointment, then the day and finally the time. We can also see embodied scaffolding when the caregiver uses her finger to point at the robot’s teeth, guiding Ida in programming the day. Ida exploits this interactive structure by looking for the relevant appointment cards and dropping them into the robot’s mouth; she presses the indicated tooth and thus demonstrates her knowledge of the programming process; she collaborates with the caregivers request and thus engages in the joint programming of the robot. During this interaction, Ida is constructed as the agent of the activity, she is the person programming the robot, while the carer is constructed as her assistant. It stays however unclear how much Ida actually remembers about the appointment and her commitment to it. In contrast to formulations such as “do you remember X″, the reminder by the caregiver is disguised in the form of instruction and information giving. Similarly, Ida’s actions do not demonstrate a ‘clear’ recognition of the reminder, as she does not display herself as noticing the information as information about an appointment. However, we can see that carer and Ida establish alignment of their activities in the joint venture of programming the robot, whereby Ida can become a competent participant of this interaction, in spite of her memory impairment (see also Goodwin, 2004).

### 3.3 Reminding as a result of a cross-situational scheduling process

Looking back at the video material, we also found situations in which the care personnel described the larger context of reminders. Reminders are not only a product of the ongoing interaction but they are also a result of a prior scheduling process. This scheduling process is essential as it indicates a person’s commitment to contribute to a future appointment. As such it has a social and moral dimension as it refers more or less explicitly to a former commitment of a person to do something, e.g., exercise for one’s own benefit, go to an appointment together with others, or water the plants for a friend. Scheduling and reminding are linked over time, where scheduling is pointing forward to a future commitment and reminding pointing backward to it, and they are build around a network of different actors.

In one workshop the care personnel describes how she agrees with Ida on appointments, e.g., going to the hair cutter, before she (the care personnel) schedules the appointment in Ida’s private and the organisational calendar, organises logistics of when, where, and how to get there, or what to bring. Afterwards she informs Ida the appointment is fixed and reminds her of it again during the morning routines on the day of the appointment. That means that reminders have an interactional and historical connection across different institutional activities with external and internal actors. Ida is excluded from large parts of activities that are relevant for the constitution of her appointments. However, the care personnel showed a strong interest in including Ida further in this process and argued strongly against our idea that the robot is linked to a calendar system or that appointments are set by a carer from a distance. Instead of just informing Ida, that an appointment has been scheduled, her primary caregiver wished to sit down with Ida and not only talk but also schedule the appointment into the robot. at *Ida's* [place] (nods).

Carer: “…if I have made an appointment with Ida to - ehm, now you will go to the hair dresser or something, then one can just type this into [the robot] here at Ida’s [place].”

This wish is based on the carers aim to include Ida as much as possible in matters that concern Ida’s life. The carer argued “*yes, because that is something Ida can do herself, so (looking at Ida), so you are free ehm from me. It is about that you can do as much as possible independently, decide on your own.*”

Similar to the programming of the robot, we can see that Ida is not constructed as a person with ‘full autonomy’. The carer’s aim is instead to enable her to participate in the process and take over smaller activities in it that are fitted to Ida’s abilities. Being part of programming the robot would enable Ida in gaining a bit more control over the routines of scheduling her appointments. The question is thus, how scheduling and reminding is organised in Ida’s everyday life and how this can inform the reminder robot to foster Ida’s participation in the process.

Therefore, we claim that we need to shift the focus from the robot’s reminder function alone to the sequential process that results in a reminder including the assistive network that supports the person with ABI. Summarizing our insight from reviewing our video-data from the co-creation process with a perspective on practices of reminding we can point out:

• During the workshops reminders were constructed as “expected next events” in the interaction of the workshop participants that display each other as waiting together for the reminder. As such, the music is framed as a reminder-signal, before it even happens.
• During the programming of the robot the required cognitive and embodied knowledge is distributed between different bodies (caregiver and Ida) and is made available for each other in the course of the interaction. Hence the interaction with the robot transfers the classical dyadic participation framework and includes the care giver as important actor in this triadic human-robot interaction (Krummheuer et al., 2020; Hornecker et al., 2022).
• The fact that Ida might not remember is not pointed out by the participants, nor does it lead to the breakdown of the interaction. We can see the performance of reminder and scheduling practices in which Ida can take part as much as possible. Hence, the aim shifts from building a robot that enables Ida’s autonomy to remember an appointment to a robot that provides a material framework that can be exploited by Ida and the staff to include Ida in the scheduling of her individual appointments *independent* of her ability to actually remember.
• Reminders have a interactional and institutional history, as they are the result of a scheduling process across different situations, actors and media.

## 4 Reminder robots in other research projects

At the time we started the project (2017) we found only a limited amount of empirical knowledge about the specific configuration of social robots in co-design with people with memory impairments (Rodil et al., 2018). In the following, we report our search for studies on reminding robots in institutional healthcare from the last few years (2016–2023). We were interested in A) the scientific perspective and research design, i.e., how the reminder robot was approached and framed by the research project and B) understanding of the reminding process and the reminder robot, i.e., what characteristics of reminding where mentioned. We restrict our search to this time frame because 1. Participatory approaches have only in recent times gained momentum in Robotics, and 2. Robotic platforms have matured to a state where they can be deployed in the field and tested on the target users. We can differentiate the literature into three categories, an overview is given in Table 1: 1. Design studies, 2. Technical developments for reminder robots, 3. Combinations, long-term evaluations and field tests.

**TABLE 1:** Types of studies and related literature.

Type of studies	Related Literature
Design studies	Casaccia et al. (2019), D’Onofrio et al. (2016), Gasteiger et al. (2022), Johnson et al. (2020), Lee et al. (2017), Lee et al. (2023), Lukasik et al. (2020), Macis et al. (2022),Moharana et al. (2019), Paletta et al. (2019), Salihs et al. (2016), Tobis et al. (2021), Tsai and Lin (2018), Winkle et al. (2018), Wu et al. (2016), Zuschnegg et al. (2021)
Technical studies	Ahn et al. (2017), Bartl et al. (2016), Bedaf et al. (2018), Casey et al. (2020), Cooper and Lemaignan. (2022), Fischinger et al. (2016), Gross et al. (2015), Koceska et al. (2019), Kostavelis et al. (2016), Mahajan and Vidhyapathi. (2017), Moro et al. (2019), Nuovo et al. (2018), Rincon et al. (2019), Saunders et al. (2016), Wengefeld et al. (2022)
Combination + evaluations	Bajones et al. (2019), Gasteiger et al. (2021), Jönsson et al. (2019), Rehm et al. (2016), Zsiga et al. (2013), Zsiga et al. (2018)

### 4.1 Design studies

In the design studies (see Table 1 first row), two main methods are employed to gain insights into users’ and stakeholders’ perspectives on the use of SARs. *Focus group* studies discuss the potential and expectations around robots with stakeholders like care personnel and target user like people with mild cognitive impairments (MCI) or dementia. *Participatory design* studies go a step further and often try to develop first designs or prototypes together with stakeholders and users. The different studies demonstrate that stakeholders and target users have different expectations and preferences regarding the reminder robots’ tasks and their acceptance.

#### 4.1.1 Focus groups

A broad range of stakeholders are participants in the different studies ranging from informal carers such as relatives (Johnson et al., 2020; Zuschnegg et al., 2021) over healthcare professionals such as nurses and clinicians (Paletta et al., 2019; Johnson et al., 2020; Zuschnegg et al., 2021) to the target users such as older people with or without MCI (Wu et al., 2016; Paletta et al., 2019; Johnson et al., 2020). While healthcare professionals and informal carers see reminders as a relevant service task for social robots in care contexts (Johnson et al., 2020; Zuschnegg et al., 2021) and point out a number of different reminding functions like activities of daily living (ADL), medication or food and drinking, target users are less positive about reminding functions for robots. Needing a robot to remember taking medication or going to activities was seen as a constant reminder about their cognitive condition and thus has a negative connotation (Wu et al., 2016).

Overall, the discussion of stakeholders’ expectations and concrete design solutions stays vague. While some interviews are more concrete naming certain activities, others identify reminders as potentially relevant. This vagueness can also be seen in the lack of concrete ideas how reminders can be transferred into technical solutions. As such, there is neither a clear idea whether a reminder is a good task for a robot, nor how the robots should do the reminder. This leaves the impression that reminders are named as “add-ons” — as potentially relevant activities that are part of everyday life in care settings they should not be forgotten when thinking of relevant tasks. This vagueness might also be a necessary uncertainty, as most projects are directed to the early stages of designing and developing processes, in which both ideas of what a technology can or cannot do are not well defined. As robots are not part of everyday life and media discourses on them are various and often more fiction than science it might also be difficult to imagine concretely what they can and should do.

#### 4.1.2 Participatory studies

While focus groups are good in identifying promising ways for development they lack specificity when it comes to concrete solutions. Participatory design studies often create more tangible results for the technical development based on the insights gained from a broad range of stakeholders like informal carers (Moharana et al., 2019), healthcare professionals (Casaccia et al., 2019; D’Onofrio et al., 2016; Lee et al., 2017; Salihs et al., 2016; Winkle et al., 2018), and target users (Casaccia et al., 2019; D’Onofrio et al., 2016; Gasteiger et al., 2022; Lee et al., 2017; Lee et al., 2023; Salihs et al., 2016). Some Studies are exploring the general use of SARs in care contexts and confirm the impression of both healthcare professionals as well as target users that reminders are viewed as suitable service tasks for such robots (D’Onofrio et al., 2016; Lee et al., 2017; Salihs et al., 2016). Others depart from the reminding function as their focus of inquiry, and develop design requirements (Moharana et al., 2019), derive new information on the realization of reminders (Winkle et al., 2018), or develop the reminders for a specific robot (Casaccia et al., 2019).

Overall, the participatory design approaches confirm the fact that reminders are seen as suitable service tasks for robots in care contexts and provide some more insight into the concrete use of reminders in care contexts. For instance, both Winkle et al. (2018) and Gasteiger et al. (2022) found that the form and reminder protocol has to be adjusted to personal preferences of the target users. Similar to the focus groups, the specification of the reminding process and its concrete realization in specific care situations remains vague.

#### 4.1.3 Other approaches

Other approaches include surveys on the opinions of using social robots in care situations or requirement analyses for developing social robots. Lukasik et al. (2020) investigated the opinions of future healthcare personnel regarding the use of social robots in care situations using the UNRAQ questionnaire (Tobis et al., 2021). Students had a positive attitude towards SARs and named reminders for food and medication as the most important function of such a robot. Due to it is nature, the study does not provide any insights into what constitutes a reminder or what the task of the robot would be in a reminding situation. Tsai and Lin (2018) propose an intelligent cognition assistant for people suffering from dementia, which has as a central task reminders for daily activities. A non-corroborated system requirement analysis is presented without any evidence that the proposed solution would work with the target user group.

### 4.2 Technical studies

The last section showed that there is a perceived need for robotic reminder systems from different stakeholders. This assumption that reminders are prototypical tasks for SARs, is often taken for granted while focusing on the technical development of robot systems but seldom realized or thoroughly tested with the target users (e.g., Fischinger et al., 2016; Kostavelis et al., 2016; Mahajan and Vidhyapathi, 2017). For the technical studies (see Table 1 second row), we distinguish three different types of studies: 1) Classical engineering with no user involvement, 2) development of reminder functionality and evaluation in lab with or without target users, and 3) development of reminder functionality and evaluation with target users in the field.

#### 4.2.1 Engineering studies

A classical engineering example is presented by Mahajan and Vidhyapathi (2017), where the task for a robot is medical reminding and delivering the drugs to the user in an Internet of Things based home system. The user is assumed to program the robot by a calendar web interface. There has been no involvement of users in the development and the system has not been tested with target users. A similar approach is described by Rincon et al. (2019), who have developed a small humanoid robot that uses emotional facial expressions to deliver reminders to the user. The prototype works like an embodied calendar system that prompts spoken reminders due to a pre-defined schedule. The reminders are given for ADL, the paper assumes that this will have a positive effect on the user’s health, e.g., going for a walk in the park. The system has not been tested with users. Koceska et al. (2019) developed a mobile tele-medicine robot and present a technical evaluation of robot navigation and movement behavior. Reminders are presented as a prototypical task for the robot and used as one of the arguments for the development of the robot. They present no further information on this aspect of the robot. Wengefeld et al. (2022) on the other hand present a robot that has as one of its functions the reminding process but test only technical aspects like it is navigation abilities.

#### 4.2.2 Lab studies

Other studies embrace reminders as suitable tasks and as an argument for developing the system, without specifying in detail, how reminders are implemented and executed. Evaluations are often run as controlled short-term lab experiments making it difficult to assess the success of the reminding service in real life contexts. Gross et al. (2015) present a mobile care robot for elderly that includes a reminder function because target users indicated in earlier conducted user studies that they want this functionality. It remains unclear, how the reminders are delivered and how they are scheduled as the user test focuses on the technical issue of navigating correctly in the dynamic environment of the user’s home. Moro et al. (2019) conduct a comparative study with a focus group of senior citizens with MCI and two different service robots. The participants interacted shortly with the different robots and were then interviewed in relation to possible tasks the robots could be useful for. One of these tasks were calendar reminders. It has to be noted though, that the interaction did not contain any reminders. Casey et al. (2020) present the robot MARIO as a personal assistant for people suffering from dementia. It includes a calendar app for activities and events and can be personalized, e.g., for medication reminders. This seems to be a standard calendar app, it is unclear who is in control of scheduling events. Relatives mention reminders as a possible useful applications when asked about the robot as an assistive device, e.g., for medication reminders, although no details are provided if this was successful or not. Also Bartl et al. (2016) embrace reminders as suitable tasks for reminder robots and test if the robot’s personality makes a difference regarding the acceptability. Although the study is performed with users from the target group (71–91 years), it is difficult to interpret the results due the de-contextualized nature of the experiment, where the robot delivers exemplary reminders in order to showcase the functionality.

#### 4.2.3 Field studies

Bedaf et al. (2018) investigate the use of the Care-o-bot as a service robot for drink reminders. The robot reminds the user to drink, accompanies him/her to the kitchen, carries the drink back to the living room and offers additional reminders, when the drink is not consumed. They tested the system on 10 participants, that were over 60, living at home and did not show signs of cognitive decline. Participants reported the robot behavior as appropriate and could see the usefulness of the scenario, but requested more complex tasks implemented in the robot. This might be an artefact of using the Care-o-bot system, as it is a large mobile robot and thus if the only service it provides is a drink reminder, this might seem inadequate for this robot. Nuovo et al. (2018) test a mobile service robot in two different living labs. One of the services included in the robot is a reminder for medication and phone calls, which can be set in a calendar-based system by either a doctor, a caregiver or the user him/herself. The robot then finds the user at the appropriate time and delivers the reminder until the user acknowledges that s/he has noticed the reminder. The system test includes the reminding functionality. The study evaluated the usability of the services as well as the user’s preference for different interaction modalities like a tablet or robot and showed that participants rated the system as useable. While most work focuses on delivering a reminder, Saunders et al. (2016) present an approach for scheduling reminders for a robot that allows users (carers, relatives, elderly) to teach the robot by giving it directives in form of rules defining pre-conditions and actions in form of if-then statements. To be able to understand the logic behind this programming of reminders, the user has to be cognitively well functioning and Saunders and others mention that the willingness to acquire the necessary knowledge cannot be expected from elderly users.

### 4.3 Combinations, long term evaluations and field tests

Even when reminders were developed based on design work with the focus group, results are often ambiguous. A food reminder system is introduced by Jönsson et al. (2019) in collaboration with focus groups from care personnel and people with dementia. They argue for limiting the system to one function and type of reminder in order to increase ease of use. While the system was developed in cooperation with the target user groups, pilot testing shows that the reminders themselves are received with mixed results. Rehm et al. (2016) follow a similar approach by focusing on one specific task for their reminding robot. In collaboration with care personnel and senior citizens with MCI, a dinner reminding robot was developed and tested in a short field deployment. While showing promising results, the limited number of cases and the short term deployment do not allow for conclusive insights. Bajones et al. (2019) conducted a field study with a SAR that delivers reminders to older people and can report that only a third of the users found the reminding function useful. The study revealed low reliability, i.e., reminders were not delivered when they should, and a frequent use of cancellations, when reminders were delivered. At the same time, delivering reminders is among the highest rated features of the robot. Gasteiger et al. (2021) developed a SAR that focuses on the reminder task and includes nine different types of reminders among others medication, bed time or wake up reminders. The robot has been developed as a mobile service robot for elderly without or with only MCI in a co-design process, ensuring that user perspectives have been taken into account throughout the development process of the system. Six residents of a residency for elderly participated in the evaluation. The robot was installed in their homes for 1 week. Participants reported that they thought that health-related reminders could be particularly useful, but for other residents that had more severe cognitive challenges. A similar effect is reported in an evaluation by Zsiga et al. (2018). Based on a study to capture user needs for a SAR (Zsiga et al., 2013), a service robot was developed that included medication and ADL reminders. A field study was done with eight participants with no cognitive impairment. The robot stayed in their home between two and four months. Results show that the reminder functions were not popular with the users, although they were ranked high by the focus groups.

### 4.4 Characteristics of reminding robots

The previous sections revealed a number of challenges with the design and development of reminder robots. We could not find a clear understanding or theoretical account of the concept of a reminder. Research on reminder robots targets a wide range of users that display large differences in their physical and cognitive abilities ranging from healthy senior citizens to people with dementia. This opens a related wide range of topics for reminders and contexts in which they are given. Often, these are mentioned in passing, the studies refer for instance to medication, ADL or exercise. But these might all be different concepts and require different types of reminders for different user groups. In most studies, it also remains unclear what a reminder is, i.e., how it is performed by the robot and how it is embedded in everyday and institutional practices. In the following, we focus on these different aspects of reminders and revisit the concepts presented in the studies.

#### 4.4.1 User groups

Four distinct user groups can be distinguished (see Table 2). The largest and most unspecific group are older people in general with no restrictions in terms of mobility or cognition. This has some obvious advantages for the development of SARs. The participants are easy to interview and test on as they intuitively understand the concept of reminders and reflect on the use and usefulness of the robot. But they are not necessarily the group that needs or wants to use a reminder robot (e.g., Gasteiger et al., 2021). Other studies work with people with MCI and we can see a more restricted use of reminders for this group, often with a focus on a single task/context for the reminder such as medication or food (e.g., Rehm et al., 2016). Similarly, we can see a focus on topics like social activities or general ADL for people with dementia (e.g., Casey et al., 2020; Salihs et al., 2016).

**TABLE 2:** Target groups for reminder robots; MCI: mild cognitive impairment.

Target user	References
older people	Ahn et al. (2017), Bajones et al. (2019), Bartl et al. (2016), Bedaf et al. (2016)^a^, Bedaf et al. (2018), Cooper and Lemaignan. (2022), D’Onofrio et al. (2016), Gasteiger et al. (2021), Gross et al. (2015), Lee et al. (2017)^a^, Lukasik et al. (2020), Macis et al. (2022), Rincon et al. (2019), Nuovo et al. (2018), Saunders et al. (2016), Wengefeld et al. (2022), Zsiga et al. (2018)
people with MCI	Gasteiger et al. (2022), Johnson et al. (2020), Kostavelis et al. (2016)^a^, Moro et al. (2019), Rehm et al. (2016), Wu et al. (2016)
people with dementia	Casaccia et al. (2019), Casey et al. (2020), Jönsson et al. (2019), Lee et al. (2023)^a^, Moharana et al. (2019)^a^, Paletta et al. (2019)^a^, Salihs et al. (2016)^a^, Zuschnegg et al. (2021), Tsai and Lin. (2018)^a^
patients	Mahajan and Vidhyapathi. (2017)^a^, Winkle et al. (2018)^a^

^a^ Denotes design studies without robot.

Thus, while not explicit in the studies, we can see that the user group has a strong influence on the topic of the reminder (see also below Table 3). What remains unclear is how the characteristics of the target user group influence other aspects of the reminding process like the design of the robot, the interaction, the delivery of the reminder to the user, or what is expected from the user. An important point is how crucial the successful delivery of the reminder and the follow-up by the user is as a success criterium (see below Section 4.4.5).

**TABLE 3:** Reminder topics.

Topic	References
medication	Ahn et al. (2017), Bedaf et al. (2016)^a^, D’Onofrio et al. (2016), Gasteiger et al. (2021), Gasteiger et al. (2022), Gross et al. (2015), Lee et al. (2017)^a^, Lukasik et al. (2020), Macis et al. (2022), Mahajan and Vidhyapathi. (2017)^a^, Nuovo et al. (2018), Paletta et al. (2019)*, Wu et al. (2016), Zsiga et al. (2018), Zuschnegg et al. (2021)
food/drink	Bedaf et al. (2018), D’Onofrio et al. (2016), Jönsson et al. (2019), Johnson et al. (2020), Kostavelis et al. (2016)^a^, Lukasik et al. (2020), Macis et al. (2022), Paletta et al. (2019)^a^, Rehm et al. (2016)
exercise	Gasteiger et al. (2021), Gross et al. (2015), Johnson et al. (2020), Paletta et al. (2019)^a^, Zuschnegg et al. (2021)
activities of daily living	Casey et al. (2020), Gasteiger et al. (2021), Gasteiger et al. (2022), Lee et al. (2023)^a^, Rincon et al. (2019), Salihs et al., 2016)^a^, Wu et al. (2016), Zsiga et al. (2018), Zuschnegg et al. (2021)
events	Bajones et al. (2019), Bartl et al. (2016), Casey et al. (2020), Kostavelis et al. (2016)^a^, Lee et al. (2017)^a^, Paletta et al. (2019)^a^, Salihs et al. (2016)^a^, Tsai and Lin. (2018)^a^
unspecified	Casaccia et al. (2019), Cooper and Lemaignan. (2022), Moharana et al. (2019)^a^, Moro et al. (2019), Saunders et al. (2016), Wengefeld et al. (2022), Winkle et al. (2018)^a^

^a^ Denotes design studies without robot.

#### 4.4.2 Reminder topics

We found five categories of recurring reminder topics (see Table 3). Three of these are health relevant topics. Medication is often mentioned by staff and users as a good topic for reminder robots but at the same time a reluctance to use is reported due to trust issues towards the system as well as safety/security. Another relevant health issue is hydration and food. Similar to medication this is a critical topic that could have a huge impact on health institutions if such a reminder system would be successful^6^. The third health-related category in this group are exercise reminders for physical as well as cognitive fitness. Other reminder topics include ADL and event notifications. Both are more tailored to social interactions but include also appointments for regular events like a visit to a doctor or the hair dresser. Lastly, there are some studies that do not mention a specific topic but talk in general about reminders.

In general, we can notice that the more mature the system becomes, i.e., the more the studies move away from exploratory methods to concrete technical realizations, the number of reminder topics becomes smaller and the focus shifts to specific reminders. This seems to indicate an inherent complexity of the reminding process that requires different interaction designs and behaviors depending on the topic or context of the reminder. Unfortunately such an in-depth knowledge about the specifics of the reminding process can rarely be found. Only Bedaf et al. (2018) acknowledge the complexity of the reminder process after focus workshops with care personnel.

#### 4.4.3 Reminder delivery

The predominant delivery form for a reminder is language-based, either as spoken verbal utterances or as text on a screen. Others give auditory calendar prompts like they are known from smartphones or utilize screens attached to the robots for visual output with pictures or videos (see Table 4). A bit surprising, the largest group are studies that do not specify how the reminder is delivered to the user, although this seems to be a crucial moment for the whole reminding process as described in Section 2.

**TABLE 4:** Reminder delivery.

Mode	References
verbal message	Bedaf et al. (2018), Casaccia et al. (2019), Cooper and Lemaignan. (2022), Gasteiger et al. (2021), Gasteiger et al. (2022), Lee et al. (2023)^a^, Macis et al. (2022), Nuovo et al. (2018), Rehm et al. (2016), Rincon et al. (2019), Saunders et al. (2016)
text message	Bajones et al. (2019), Tsai and Lin. (2018)^a^
visual (picture/video)	Ahn et al. (2017), Cooper and Lemaignan. (2022), Jönsson et al. (2019), Lee et al. (2023)^a^, Salihs et al. (2016)^a^
calendar prompt (audio)	Bartl et al. (2016), Mahajan and Vidhyapathi. (2017)^a^, Paletta et al. (2019)^a^
unspecified	Bedaf et al. (2016)^a^, Casey et al. (2020), D’Onofrio et al. (2016), Gross et al. (2015), Johnson et al. (2020), Kostavelis et al. (2016)^a^, Lee et al. (2017)^a^, Lukasik et al. (2020), Moharana et al. (2019)^a^, Moro et al. (2019), Wengefeld et al. (2022), Winkle et al. (2018)^a^, Wu et al. (2016), Zsiga et al. (2018), Zuschnegg et al. (2021)

^a^ Denotes design studies without robot.

We also noticed an under-utilization of the embodied qualities of a robot. Most reminder deliveries use either single modalities or additional mediums like a tablet for delivering the reminder. One of the advantages of robots is their embodiment and physicality, which allows for more natural interaction making use of gaze, gestures, body movements, etc. for communication purposes. This is highlighted in a recent paper with a focus group of people with dementia that mention the multimodal delivery of reminders as one advantage of robots (Lee et al., 2023). Current system design though, seems to treat reminders more or less as calendar functions and do not develop an understanding of the physical interaction involved in delivering the reminder. There seems to be an untapped potential here, where the embodiment of the robot could determine the successful delivery of the reminder including aspects such as how (and when) the robot approaches the user, how the robot engages the user in the reminding action, or how the robot directs attention to the reminding object, e.g., for a drinking reminder.

#### 4.4.4 Robot behavior

Most approaches aim at finding the user at the right time and then delivering the reminder through the means presented in the previous section (see Table 5). Only few studies monitor the success of the reminder and/or repeat the reminder if necessary, and only one exploratory study acknowledges the complexity of the reminding delivery and the need to adapt the reminder to the situation. Similar to the delivery of the reminder, many studies do not specify the robot behavior for the reminder. We assume that this is due to the observation that reminders are seen as prototypical scenarios but seldom realized and tested.

**TABLE 5:** Robot behavior.

Behavior	References
move to/find user	Bedaf et al. (2018), Cooper and Lemaignan. (2022), Gross et al. (2015), Lee et al. (2023)^a^, Moharana et al. (2019)^a^, Nuovo et al. (2018), Saunders et al. (2016), Winkle et al. (2018)^a^
present reminder at appropriate time	Ahn et al. (2017), Bajones et al. (2019), Bedaf et al. (2018), Cooper and Lemaignan. (2022), Gross et al. (2015), Kostavelis et al. (2016)^a^, Lee et al. (2023)^a^, Macis et al. (2022), Moharana et al. (2019)^a^, Nuovo et al. (2018), Rehm et al. (2016), Rincon et al. (2019), Salihs et al. (2016)^a^, Saunders et al. (2016), Tsai and Lin. (2018)^a^, Winkle et al. (2018)^a^
repeat reminder when necessary	Casaccia et al. (2019), Lee et al. (2023)^a^, Macis et al. (2022), Rehm et al. (2016), Winkle et al. (2018)^a^
monitor success	Casaccia et al. (2019), Lee et al. (2023)^a^, Nuovo et al. (2018)
adapt reminder to situation	Bedaf et al. (2016)^a^
unspecified	Bartl et al. (2016), Casey et al. (2020), D’Onofrio et al. (2016), Gasteiger et al. (2021), Gasteiger et al. (2022), Jönsson et al. (2019), Johnson et al. (2020), Lee et al. (2017)^a^, Lukasik et al. (2020), Mahajan and Vidhyapathi. (2017)^a^, Moro et al. (2019), Paletta et al. (2019)^a^, Wengefeld et al. (2022), Wu et al. (2016), Zsiga et al. (2018), Zuschnegg et al. (2021)

^a^ Denotes design studies without robot.

From the implemented robot behaviors, we can see that the reminding process is in most cases conceptualized as a simple delivery task, where the robot finds the user at the right time and delivers a notification about the reminding topic. Success criterion seems to be to deliver the reminder at the right time to the right person. Implicit in this is a specific expectation about user compliance, i.e., that the user just needs the notification to remember what (and why) to do the notified activity. For most of the proposed target groups (people with MCI or dementia) this is an optimistic assumption, as we also discovered in our case study (see Section 2).

#### 4.4.5 User expectations

In the last section it became apparent that there is a clear expectation that the user complies with the reminder and does the required action. This can be seen in the studies that actually investigate this aspect of the reminding process (see Table 6). This is a critical problem as we have seen in our case study (see Section 2). Crucially, most studies do not specify any expectations towards the user behavior and do not consider what happens after the user has been reminded. It can be assumed, that also in these cases the user is expected to comply and do the required action.

**TABLE 6:** Expectation from user.

Expectation	References
do relevant action	Ahn et al. (2017), Bedaf et al. (2018), Jönsson et al. (2019), Nuovo et al. (2018), Rehm et al. (2016), Tsai and Lin. (2018)^a^
unspecified	Bajones et al. (2019), Bartl et al. (2016), Bedaf et al. (2016)^a^, Casaccia et al. (2019), Casey et al. (2020), Cooper and Lemaignan. (2022), D’Onofrio et al. (2016), Gasteiger et al. (2021), Gasteiger et al. (2022), Gross et al. (2015), Johnson et al. (2020), Kostavelis et al. (2016)^a^, Lee et al. (2017)^a^, Lee et al. (2023)^a^, Lukasik et al. (2020), Macis et al. (2022), Mahajan and Vidhyapathi. (2017)^a^, Moharana et al. (2019)^a^, Moro et al. (2019), Paletta et al. (2019)^a^, Rincon et al. (2019), Salihs et al. (2016)^a^, Saunders et al. (2016), Wengefeld et al. (2022), Winkle et al. (2018)^a^, Wu et al. (2016), Zsiga et al. (2018), Zuschnegg et al. (2021)

^a^ Denotes design studies without robot.

We have seen in our example that receiving a reminder is not a trivial task. In general, the reminder is supposed to trigger the remindee’s memory of the reminding topic. But first the remindee needs to identify the reminder as a reminder, otherwise it is “noise” with no apparent meaning. Then the remindee needs to connect the reminder to a suitable action. The complexity of this part depends on the topic and can range from single actions like taking the medication over simple action sequences like getting to the kitchen, fetching a drink and drinking it, to complex action sequences that are distributed over a longer time frame like getting ready for an appointment at a different place, leaving the apartment and going to the appointment, and then attending the appointment. This is quite complex for the envisioned target groups of reminder robots, thus it seems to be necessary to focus on specific reminders and take into account how crucial the successful follow-up by the user is. For instance, not complying with a reminder for a social activity could be seen as less severe than not complying with a reminder for taking a necessary medication.

#### 4.4.6 Scheduling

Before the robot can remind the user, the reminder has to be scheduled. We can distinguish five categories of responsibles for scheduling of reminders (see Table 7). In some cases, the user is expected to schedule reminders. In institutional settings it is more appropriate that caregivers (either formal or informal) or medical personnel schedule the reminders because the user will not have all the information necessary about free appointments, care schedules, etc. In one case, the researcher did the scheduling for a prototype. Again, a number of studies do not take the scheduling into account as an inherent feature of the reminding process.

**TABLE 7:** Responsible for scheduling reminder.

Scheduling	References
user	Bajones et al. (2019), Casaccia et al. (2019), Gasteiger et al. (2021), Mahajan and Vidhyapathi. (2017)^a^, Nuovo et al. (2018), Tsai and Lin. (2018)^a^, Wengefeld et al. (2022)
care personnel (formal caregiver)	Jönsson et al. (2019), Macis et al. (2022), Nuovo et al. (2018), Rehm et al. (2016), Salihs et al. (2016)^a^, Saunders et al. (2016)
relative (informal caregiver)	Bajones et al. (2019), Casaccia et al. (2019), Saunders et al. (2016)
medical personnel (doctor, nurse, pharmacist)	Ahn et al. (2017), Nuovo et al. (2018)
researcher	Bartl et al. (2016)
unspecified	Bedaf et al. (2016)^a^, Bedaf et al. (2018), Casey et al. (2020), Cooper and Lemaignan. (2022), D’Onofrio et al. (2016), Gasteiger et al. (2022), Gross et al. (2015), Johnson et al. (2020), Kostavelis et al. (2016)^a^, Lee et al. (2017)^a^, Lee et al. (2023)^a^, Lukasik et al. (2020), Moharana et al. (2019)^a^, Moro et al. (2019), Paletta et al. (2019)^a^, Rincon et al. (2019), Winkle et al. (2018)^a^, Wu et al. (2016), Zsiga et al. (2018), Zuschnegg et al. (2021)

^a^ Denotes design studies without robot.

From the range of reminding topics (see Table 3), we argue that depending on the topic scheduling has to be done by different stakeholders and might warrant a rather complex back-end for the reminding robot. For instance, medication reminders should be set by medical personnel or formal care givers, whereas food and drinking reminders could be set by both formal and informal care givers. This creates some interesting technical challenges regarding privacy and data protection for the system, an aspect that has not been investigated in any of the studies.

## 5 Discussion

The previous sections have reviewed a substantial amount of work focusing on reminders as a function for SARs. While reminding is often described as a prototypical task for socially assistive robots in healthcare contexts and although reminding is often viewed as an intrinsic element of SARs as we have seen above, it is rarely explicitly modelled. Typical topics for reminders are medicine, food/drinking, and ADL, where is has to be noticed that the kind of activity is often unspecified.

Reminders are seen as isolated events that have to be delivered on time and then are supposed to trigger the relevant memory and behavioral processes in the user. This is seldom made explicit. The expectations towards the user as a reaction to the reminder are most of the time unspecified with the underlying assumptions that the user will comply with the reminder once it has been issued by the robot, i.e., the user will remember what to do triggered by the reminder. A little surprising is that the specific behavior of the robot delivering the reminder is often unspecified, making it difficult to imagine how the robot will be able to deliver the reminder. When specified, the delivery itself is often implemented as a verbal or visual notification and the question how the robot knows about the user’s activities is either not considered or delegated to the care personnel or informal carers that schedule the reminders.

**FIGURE 3:**
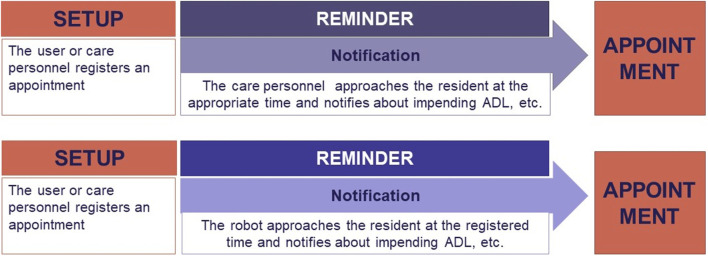
Reminding without and with SARs.

The reminding process derived from this analysis, and the one that we assumed in our first design of Ida’s reminder robot, can be seen in Figure 3. The main ingredient of the reminding process is the isolated reminding incident, where the care personnel reminds the person about an upcoming event assuming that the person then will remember this event and act accordingly (Figure 3 above). The setup of the reminder takes place by placing the event in a calendar system, which is either done by the person self or the care personnel. In this process, it becomes relatively straightforward to integrate a robot to take over this specific task of notifying the user with the unspoken assumption that the robot will be able to blend easily into the care context (Figure 3 below).

**FIGURE 4:**
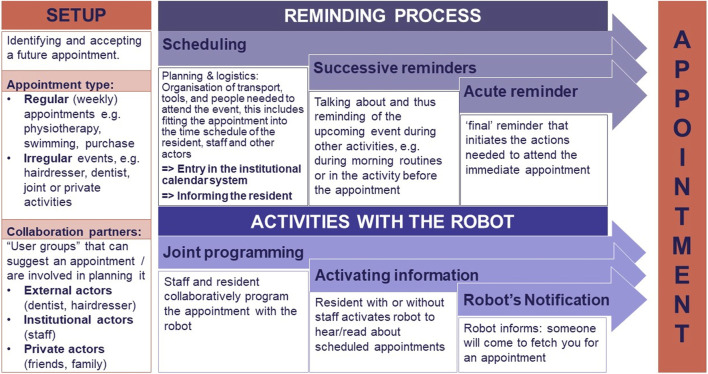
Process model of reminding practice.

While sounding trivial, providing relevant reminders is a contextually rich and often individual task. As we have seen from our ethnographic description, successful reminders in the interactions with Ida are embedded in a) situated and collaborative processes in which the reminder is produced as an interactive achievement, and b) we can observe an interactional history of reminders that is framed in organisational routines of reminding as described in the process model of reminding practice (Figure 4). This complexity is mentioned only by a few studies like Amrhein et al. (2016), who emphasise the supportive network that frames the scheduling and reminding of activities in interaction with people with special needs. In their ethnographic design study they differentiate three steps: 1) appointment registration, 2) reminding practice, in which the staff reminds the client of an appointment, 3) the appointment itself. Bedaf et al. (2016) present their design workshops with older people and their support staff where reminders are investigated as a suitable task for a SAR. During their workshops it became apparent that while reminders might be suitable tasks for SARs, the task of reminding is far from trivial. Care personnel take into account contextual and individual characteristics when providing reminders and always phrase reminders to acknowledge independence, i.e., reminders are provided for things people want to be reminded of and they should not become irritating.

Thus, reminders are complex practices that are relying on contextual and individual factors and are created in the situated interaction between reminder and remindee. In Figure 4 we provide a model for this complex reminder process that could guide future projects of reminder robots.

Before the appointment is registered into an institutional calendar system, the appointment has to be identified and mutually agreed on by the carer, the resident and potential external actors like the dentist or the hair dresser. We call this phase the *setup*, which indicates different interactions and activities that take place when people identify and accept a future appointment. We differentiate between *regular weekly appointments*, such as physiotherapy, swimming or shopping that have a rather predetermined time, place and participation framework, and *irregular appointments* such as going to the hair dresser, dentist or participating in joint activities (e.g., a tour to the cinema). Some of these appointments are determined by others, e.g., when the dentist calls for the yearly check, or when the physiotherapist schedules weekly training sessions. In our case for instance, the physiotherapist follows an agreement that Ida should have 2 sessions a week, but the time could change, depending on the other residents he is working with. Shopping is mostly done on a certain day of the week, but the time is often adapted to the working schedule of the caregiver who accompanies Ida. Thus, Ida’s individual preferences are negotiated with the institutional schedule.

All appointments have to be mutually agreed on between residents and staff that is responsible for fitting them into the institutional calendar along with the resident’s personal schedule. The latter can be compared to the appointment registration described in Amrhein et al. (2016). Along with this registration, the staff organises the logistics to realise the appointment, which means organising transport, personnel or tools (e.g., remember to pack the swimming suit), which are necessary to attend the appointment. In our case, the planing of the appointment is often adjusted to Ida’s individual preferences, e.g., taking into account her naptimes, her energy-level during the day or that Ida likes to be accompanied to shopping by a certain person. Ida is informed when a appointment is scheduled, the task of planning it (in collaboration with others), however, lies in the hands of the personnel.

When the actual appointment approaches, staff introduces the upcoming event during their conversation with the resident, e.g., during the morning routines. This reminding activity can be compared to what Amrhein et al. (2016) describe as *successive reminders*, which is a set of recurring reminders of an appointment over a certain period of time. They provide a reliability in planning activities as they support the temporal orientation of the citizen and thus prepare him/her to anticipate *acute reminders*, which are given close to the actual appointment and are often formulated as a request. Different from the analysis in Amrhein et al. (2016), the reminders in our cases do not aim for Ida to attend the appointment individually. Rather, the staff aims to help her to get an idea of what will happen today. As such they do not have a recurrent and instructive pattern, but rather an informative function for giving the residents a temporal orientation of the day. As Bedaf et al. (2016) points out, staff adapts the reminders to each individual, which would be similarly necessary for a SAR. When Ida needs to prepare and leave for an appointment, the staff enters her apartment and helps her with these activities and accompany her to the appointment.

The situated description of doing reminding and the mapping of reminding process demonstrates that reminders are much more than just a trigger-response reaction. We can see how reminding in situations with people with memory impairments is organised and co-constructed in interactive processes that have an ‘interactional history’.

## 6 Conclusion

In this paper we reflected our co-creation process of a reminder robot and presented a process model of reminding practice that takes the situated and collaborative complexity of reminding into account and moves away from viewing reminders as a simple notification of an upcoming event with the assumption that the reminded person will remember the appointment and comply to the necessary action. From our reflections we can formulate the following criteria for the development of reminder robots for people with memory impairment:

• *The relevance of practice over discussion:* We propose a co-creation process that includes all relevant participants. However this design process needs to have iterations focusing on the analysis and reflection of practices. We would suggest a combination of mapping of practices as, e.g., suggested in Crabtree et al. (2012) and collaborative video data analysis as suggested in Lucero et al. (2016).
• *Reminders cannot be understood as isolated events:* Assistive reminding practices are the result of an process in which the reminder is prepared. Understanding reminders as part of a process, allows us to understand how it is related to former and later activities (e.g., committing to an activity at a certain point of time, pre-reminders and the acute reminder). Understanding reminding as a process across time and places, allows for a deeper inclusion of persons with memory impairment in reminder processes supported by both human and technical assistance. We therefore claim that during the development of SARs, the whole process of reminding has to be taken into account in order to achieve as much independence as possible for the person with memory problems.
• *From independence to participation:* Reminders demand complex cognitive abilities on the side of the remindee. The competences to understand a reminder and its consequences can be too demanding for the envisioned target group of reminder robots. We suggest a change from technologies that aim for (full) autonomy to technologies that aim for meaningful participation of people with memory impairments. This means to take into account their communicative and embodied communication resources and establish technical and interactive frameworks that enable their participation as competent participants.
• *The realization of reminders has to be adapted to individual users.* Such a “personalization” has to include relevant reminder topics, the modes of reminding, the interaction design for scheduling, the behavior of the robot and careful adjustments of reminders towards the user’s daily rhythm, situated mood, and personal preferences. Instead of a “one reminder fits all” solution, SARs need to reconsider the ways reminders can be individually adjusted.
• *Non-dyadic interaction:* As pointed out elsewhere (Hornecker et al., 2022), especially in healthcare context human-robot interaction goes beyond the classical assumption of a dyadic user-robot interaction. Instead we can often see other actors facilitating, moderating or just watching the interaction with the robot. The role of this co-participants needs to be much more considered in HRI for real-life contexts (see also Krummheuer et al., 2018).

We would like to end with a call for more empirical research on reminder practices and robots. Looking at the current research on reminding and reminder robots, we need much more systematic and empirical knowledge on concrete embodied and material practices and processes of reminding in social lives of people with memory impairments and their carers.

## Data Availability

The datasets presented in this article are not readily available because video recordings of interactions with vulnerable participants cannot be shared. Requests to access the datasets should be directed to antonia@ikp.aau.dk.

## Footnotes

1 see https://center-tbi.eu, https://www.hjernesagen.dk/om-hjerneskader/facts-om-hjerneskader and https://hjerneskadet.dk/viden-om-hjerneskade/hjerneskader-i-tal/(24 March 2024).

2 see https://videnscenterfordemens.dk/da/tal-og-statistik, https://www.alzheimer.dk/presse-politik/fakta/demens-i-tal/and https://alzheimer-europe.org (24 March 2024).

3 Not her real name.

4 While seemingly inconsistent from a HRI perspective, this combination of modalities was explicitly chosen by the participants to ensure training of different movements while working with the robot.

5 This event made a lasting impression on the care personnel. Even though we agreed to collaboratively re-design the robot’s reminder in further workshops the care personnel denied all solutions with sound from that day on. Ida on the other hand could not remember the troubles caused by the robot.

6 According to a report from the Danish Health Ministry dehydration is responsible for over 10% of unnecessary hospitalizations in the age group above 65 and over 15% for the age group above 85 (Ministeriet for Sundhed og Forebyggelse, 2015).
